# Opto Field-Effect Transistors for Detecting Quercetin–Cu^2+^ Complex

**DOI:** 10.3390/s22197219

**Published:** 2022-09-23

**Authors:** Pradhana Jati Budhi Laksana, Li-Chu Tsai, Chang-Cheng Lin, Kuei-Shu Chang-Liao, Mathew K. Moodley, Chii-Dong Chen

**Affiliations:** 1 Department of Engineering and System Science, National Tsing Hua University, Hsinchu 30013, Taiwan; 2 Nano Science and Technology Program, Taiwan International Graduate Program, Academia Sinica, Taipei 11529, Taiwan; 3 Institute of Physics, Academia Sinica, Taipei 11529, Taiwan; 4 Institute of Organic and Polymeric Materials, National Taipei University of Technology, Taipei 10608, Taiwan; 5 Discipline of Physics, School of Chemistry and Physics, College of Agriculture, Engineering and Science, University of KwaZulu-Natal, Durban 4000, South Africa

**Keywords:** molecular absorption spectrum, molecular charge, field-effect transistor, optical, quercetin, metal complexes, copper ion

## Abstract

In this study, we explored the potential of applying biosensors based on silicon nanowire field-effect transistors (bio–NWFETs) as molecular absorption sensors. Using quercetin and Copper (Cu^2+^) ion as an example, we demonstrated the use of an opto–FET approach for the detection of molecular interactions. We found that photons with wavelengths of 450 nm were absorbed by the molecular complex, with the absorbance level depending on the Cu^2+^ concentration. Quantitative detection of the molecular absorption of metal complexes was performed for Cu^2+^ concentrations ranging between 0.1 μM and 100 μM, in which the photon response increased linearly with the copper concentration under optimized bias parameters. Our opto–FET approach showed an improved absorbance compared with that of a commercial ultraviolet-visible spectrophotometry.

## 1. Introduction

Many trace metal ions are essential to the human body and play an important role in human health. In small quantities, certain heavy metals such as copper (Cu^2+^), manganese (Mg^2+^), and zinc (Zn^2+^) with concentration of 25 μM, 7 μM, and 430–650 μM, respectively, have a substantial impact on our health. Above that tolerance concentration, heavy metals are harmful and can lead to various disease conditions [1]. Among these ions, the complex stability of quercetin and copper (Cu^2+^) ion is reported to be the highest [2], and metal chelation in quercetin–Cu^2+^ ion has been intensively studied in recent years [3,4,5,6,7]. These studies were performed using optical spectroscopy such as infrared spectroscopy [8,9], ultraviolet–visible (UV–Vis) spectrophotometry [10,11], and electrospray ionization mass spectroscopy [2]. Optical spectroscopy is based on the idea that the absorption of the quercetin–Cu^2+^ complex varies at different wavelengths, depending on its complex electronic structures. Therefore, spectroscopy is a powerful tool for observing molecular complexes and structures, and thus, for observing the chemical bonding in the excited state of quercetin–Cu^2+^ ion complexes. As reported in the literature, using UV–Vis spectrophotometry, quercetin showed band I absorption (~300–380 nm) and band II absorption (240–280 nm). An absorption peak at 370 nm observed upon the reaction with copper ion at a ratio of 1:1 shifted to approximately 450 nm upon increasing the copper ion concentration [9,12]. In this study, we propose the use of a silicon nanowire field effect transistor (bio–NWFET) platform to study the shift and to detect various concentrations of copper based on quercetin–metal ion interactions. Bio–NWFET consists of dual gates. Both gates can modulate the carrier concentration of source-drain channels. The front gate is used as a sensing area that is sensitive to physical stimuli, and the back gate has the advantage of adjusting and obtaining complex gate controllability for keeping the typical transistor behavior. It enables the improvement of sensing performance of the devices [13,14,15].

Bio–NWFET has been reported as a powerful platform for the detection of interactions between bio-molecules [16,17]; however, the use of bio–NWFET in an FET’s optoelectronic function to form a dual-function sensing device is lesser known [18]. The function has the unique advantages of mass-production semiconductor device technology, miniaturization, low power consumption, and ease of integration [19]. In our previous study [18], we integrated the charge sensing and optical transduction functions of bio-NWFETs for the detection of antibody–antigen interactions using ELISA technology. In this study, we employed quercetin and Cu^2+^ ion as molecules for detecting the molecular absorption of metal complexes. Silicon offers an absorption range from ultraviolet to visible wavelengths, owing to its high bandgap energy of 1.1 eV. We investigated the effect of internal factors such as drain voltage (V_DS_) and back-gate voltage (V_BG_) and external factors such as intensity and wavelength on the charge carrier to obtain the optimum linearity for the detection of copper based on ion and quercetin interactions. Based on these parameters, we demonstrate the measurement of quercetin and Cu^2+^ interaction. Further, the absorption results in different buffer solutions of 0.01X, 0.1X, and 1X were compared with those obtained using UV–Vis spectrophotometry.

## 2. Materials and Methods

### 2.1. Reagents and Chemicals

The following reagents were used: quercetin (C_14_H_10_O_7_) 99% purity from Acros Organic, copper(II) chloride (CuCl_2_) 99% purity from Sigma-Aldrich (St. Louis, MI, USA), and Dulbecco’s phosphate buffered saline (10X PBS, 137 mM NaCl, 2.7 mM KCl, 10 mM Na_2_HPO_4_, 2 mM KH_2_PO_4_, pH 7.4) from Invitrogen.

### 2.2. Detection Procedure

The concentration of quercetin stock solution was 1 mM, prepared in a methanol solution, and diluted to 100 μM using a PBS solution. A 100 μM quercetin solution was added to 0.1, 0.5, 1, 10, 20, 40, 80, and 100 μM copper chloride (CuCl_2_) and reacted in 1X PBS solution for a period of time. Spectra were obtained on a commercial optical density (OD) spectrometer (JASCO V670). In our opto–FET, measurements were conducted in the system over several on/off cycles for each concentration of CuCl_2_. PBS solutions were injected in between different tested samples to wash the fluidic channel and make sure there is no leftover sample during measurement.

### 2.3. Measurement Setup

**Figure 1:**
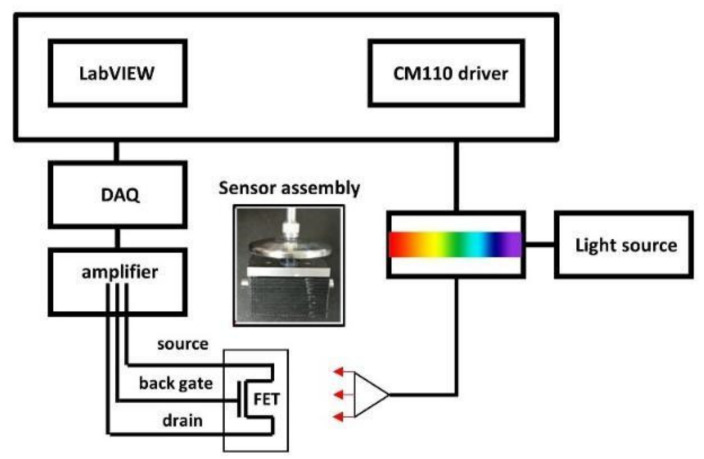
The block diagram and photographs of the measurement system.

Electrical measurements of the NW-FET device were obtained using a homemade data acquisition system connected to a computer running software written using LabVIEW (National Instruments, Austin, TX, USA), as shown in Figure 1. The sensor assembly accommodated an NW-FET chip with fluidic channels and an optical fiber adapter, and was connected to the acquisition platform using an HDMI cable. The specimen solution was injected into the device sensing area through a fluidic channel with a sample of approximately 14.1 μL; the solution inlet and outlet were located at the two sides of the sensor assembly. The length, width, and thickness of the fluidic channel were 7.34 mm, 1.2 mm, and 1.6 mm, respectively. The light source consisted of a xenon fiber optic light source (ASB–XE–175EX, Spectral Products) and a monochromator (CM110, Spectral Products) with controlled power intensity and wavelength. Light was introduced onto the NW–FET sensing area from the top of the sensor assembly on a dark, black box at a distance of 10 mm. In this acquisition system, the user controls the measurement parameters such as the source–drain voltage (V_DS_), back–gate voltage (V_BG_), light intensity, wavelength, and time at room temperature. The light intensity of the source was calibrated using a commercial silicon photodiode, PH–100Si, from Gentech EO, Inc. (Quebec City, QC, Canada).

## 3. Results and Discussion

**Figure 2:**
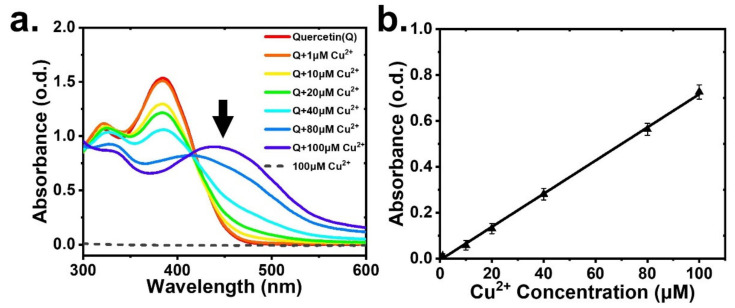
(**a**) UV–Vis absorption spectra of quercetin and various concentrations of Cu^2+^ in 1X PBS showing two peaks at 380 nm and 450 nm, and (**b**) the absorbance as a function of Cu^2+^ concentration at 450 nm.

To examine the complex of quercetin and Cu^2+^ ion in a buffer solution of 1X PBS, we used a commercial optical density (OD) spectrometer (JASCO V670) at a range of 300–600 nm, as shown in Figure 2a. The results showed no observable absorption peak for 100 μM of Cu^2+^, whereas the absorption peak for quercetin appeared at 385 nm. In the combined quercetin and Cu^2+^ solution, with increasing Cu^2+^ concentration from 1 μM to 100 μM, the absorbance peak at 385 nm decreased and even vanished; however, the absorbance at 450 nm increased and formed a peak at a Cu^2+^ concentration of 100 μM. As shown in Figure 2b, the absorbance linearly increased with the Cu^2+^ concentration at a wavelength of 450 nm, and the solution color of the quercetin–Cu^2+^ complex in 1X PBS buffer changed from transparent (colorless) to yellowish, as shown in Figure S2 in the Supplementary Materials.

**Figure 3:**
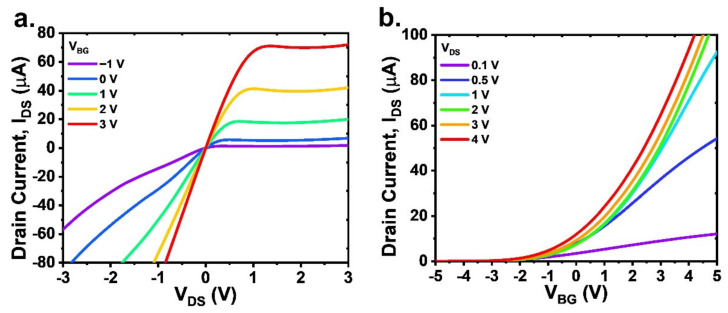
Electrical characteristics of NW-FET (**a**) transfer characteristics (I_DS_–V_DS_) at various V_BG_ from −1 V to 3 V and (**b**) output characteristics (I_DS_–V_BG_) at various V_DS_ from 0.1 V to 4 V.

The scanning electron microscopy (SEM) images with a channel length, width, and height of 2 μm, 200 nm, and 100 nm, respectively, are shown in the Supplementary Materials Figure S1. A back-gated NW-FET was fabricated using top–down techniques without a top metal gate (discussed in the Supplementary Materials on device fabrication). The NW-FET was designed to operate in accumulation mode, meaning that the device was set up with a small drain current (I_DS_) at zero gate voltage. This is an advantage for photodetectors because a high illumination intensity is not required. The device characterization was initially performed in the dark to extract the electrical properties of the NW-FET, which shows n-type characteristics, as presented in its transfer characteristics (I_DS_–V_DS_) and output characteristics (I_DS_–V_BG_) in Figure 3a,b.

**Figure 4:**
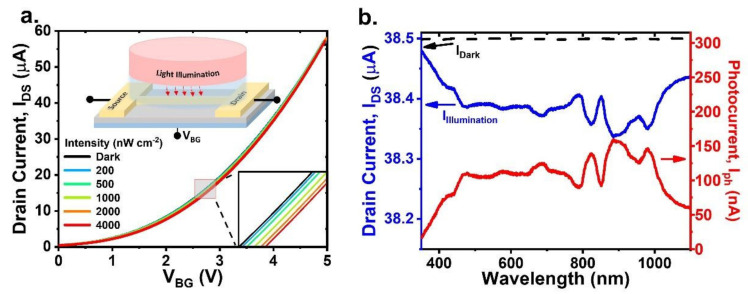
(**a**). I_DS_–V_BG_ of an opto-FET device with V_DS_ = 0.5 V, λ = 450 nm in the dark (black) and under light illumination of 200 (blue), 500 (green), 1000 (light green), 2000 (orange) and 4000 (red) in unit of nW cm^−2^. and (**b**) the photo-response of the device captured under illumination of the wavelength ranging between 300 nm and 1100 nm. The dark current (dashed black), illumination current (blue) and photocurrent (red). The spectrum is taken with V_DS_ = 0.5 V, V_BG_ = 4 V and intensity is 500 nW cm^−2^. Inset in (**a**), the schematic diagram of the opto-FET under light illumination.

Figure 4a shows a schematic illustration of the opto-FET setup, in which the photon response involves several mechanisms such as photon absorption, carrier photogeneration, and carrier transport. In addition, Figure 4a shows the transfer characteristics of the device, indicating that at a wavelength of 450 nm, the I_DS_ decreases with increasing light intensity at all values of V_BG_. The blow-up view shown on the bottom right of Figure 4a demonstrates the changes in intensity. In Figure 4b, we show the response of the I_DS_ for illumination at varying wavelengths, implying that the opto-FET responds to a broad range of wavelengths. The dark current I_dark_ is indicated in the plot to show that the I_DS_ under illumination, I_illumination_, is lower than I_dark_ in the wavelength range of interest. Many factors affect the polarity of the photocurrent, such as the photon wavelength, intensity, type of channel doping and concentration, and feasible device design [20,21]. However, the magnitude of the photocurrent, rather than the polarity, is relevant for the application of photodetectors. Therefore, the photocurrent is defined as the absolute change in the I_DS_ upon illumination, that is, I_ph_ = |I_illumination_ − I_dark_|, as shown in the same figure.

**Figure 5:**
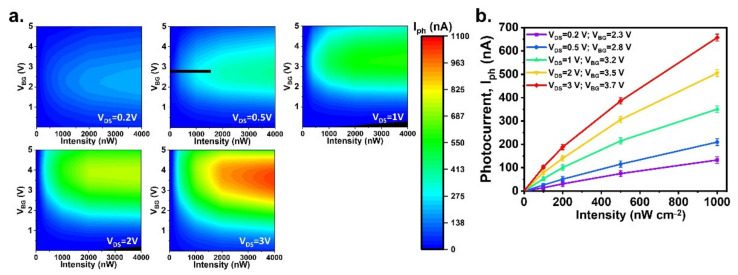
(**a**) The photocurrent at a wavelength of 450 nm plotted as functions of V_BG_ and light intensities, for V_DS_ at 0.2 V, 0.5 V, 1 V, 2 V, and 3 V. The color scale bar is shown on the right. (**b**) The photocurrent as a function of light intensity. It shows a linear dependence at V_DS_ 0.5 V and V_BG_ = 2.8 V.

Because the quercetin–Cu^2+^ complex generates a linear and strong absorption at wavelengths of 430–450 nm, a wavelength of 450 nm was chosen for use in this study. To use the opto-FET system as a photodetector for biosensing applications, a suitable bias condition should be determined, wherein the detection has good sensitivity and shows a linear dependence on light intensity. This linear dependence would enable the calibration of the molecule concentration. To this end, we measured the device by varying V_DS_ and V_BG_ at a wavelength of 450 nm under various light intensities, and the results are shown in Figure 5a. The figure demonstrates that increasing the V_DS_ value increased the photocurrent of the device. However, for different V_DS_ values, the maximum photocurrent appeared at different V_BG_ values in the range of 2–4 V. We limited the light intensity to 1000 nWcm^−2^, as intense light could harm the molecules. Figure 5b shows the intensity dependence of the photocurrent for the five V_DS_ values used in Figure 5a; in addition, the V_BG_ values corresponded to the maximum photocurrent at 1000 nWcm^−2^ for each V_DS_ trace. The plot demonstrates that the optimal bias condition for this opto–FET was V_DS_ = 0.5 V and V_BG_ = 2.8 V.

**Figure 6:**
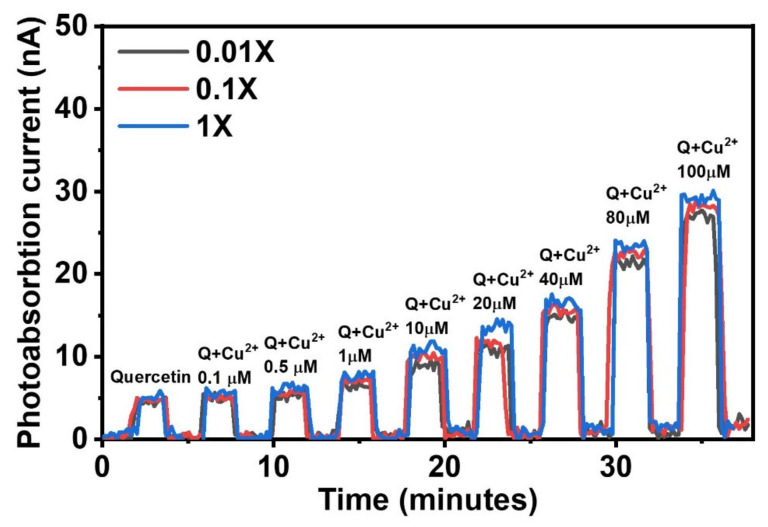
Absorption measurement of quercetin–Cu^2+^ ion mixture for various Cu^2+^ concentrations ranging from 0.1 μM to 100 μM in three different concentrations of PBS solution. A 100 μM quercetin was used as a control sample.

To illustrate the application of the opto–FET approach for the detection of the quercetin–Cu^2+^ complex, we set the light intensity to a low value of 500 nWcm^−2^. The solutions were initially mixed in a tube before being injected into the opto-FET fluidic channel. The measurements were performed over several on/off cycles controlled by a light shutter. For quantitative evaluation, various Cu^2+^ concentrations were tested at different PBS concentrations, as shown in Figure 6. Quercetin was used as the control sample, as it could absorb 450 nm photons. As expected, the photoabsorption current increased with increasing Cu^2+^ concentration.

**Figure 7:**
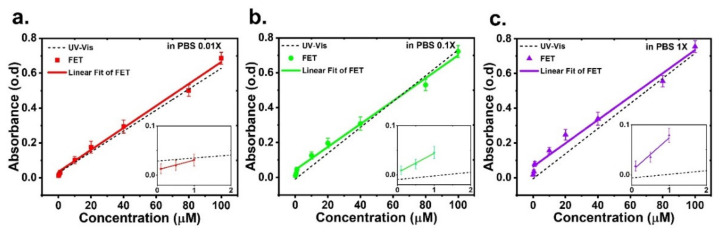
A comparison between the absorbance obtained with our opto-FET system (solid lines) and with a commercial UV–Vis spectrometer (dot-dashed lines) in PBS concentration of (**a**) 0.01X (**b**) 0.1X, and (**c**) 1X. The evaluation was performed at a wavelength of 450 nm and light intensity at 500 nWcm^−2^, V_DS_ = 0.5 V, and V_BG_ = 2.8 V.

The relation between the absorbance A of the solution and the absorption molecule concentration is described by the Beer–Lambert law [22]:

(1) $$A=-log\left(\frac{I}{I_{0}}\right)=\epsilon \cdot l\cdot c$$

where $I_{0}$ and I denote the initial light intensity and the light intensity after passing through the solution, respectively, ϵ is the molar absorptivity with the unit of L mol^−1^·cm^−1^, l is the photon path length with the unit of cm, and c is the concentration of absorbing molecule with units of mol·cm^−1^. The equation indicates that the number of molecules in the radiation path increases with the length of the path, increasing the absorbance as a result. For a given path length, absorbance is directly proportional to the analyte concentration. This relationship enabled quantitative measurement of the concentration of absorbing molecules. Using Equation (1), we calculated the absorbance of the molecule. As shown in Figure 5b, the transmittance photocurrent ($I_{0}$) was approximately 100 nA for a light intensity of 500 nWcm^−2^. The photoabsorption current ($I_{0}–I$) was obtained after passing through the solution, as shown in Figure 6. Together, we obtained the absorbance of the quercetin–Cu^2+^ complex, as shown in Figure 7. The different buffer solutions affected the wavelength during the quercetin–Cu^2+^ ion activity. Further, the absorption at a wavelength of 450 nm decreased as the buffer concentration decreased due to changes in pH value when the buffer solution was diluted. As reported in the literature [5], pH affects absorption because protons are released when the buffer forms a complex with metal ions.

As described in the measurement setup, the fluidic channel in our system was 1.6 mm thick, which was the path length of the sample. Compared with a commercial UV–Vis spectrometer, which has a path length of approximately 10 mm, the sample measured in our system was approximately 6.25 times smaller than that in a UV–Vis spectrum. The magnitude of absorbance in our system was multiplied by 6.25 times for a comparison between our opto–FET sensor and a commercial UV–Vis. We plotted the absorbance as a function of Cu^2+^ concentration, as shown in Figure 7, along with the absorbance results obtained using a commercial UV–Vis spectrometer in different buffer solutions. With the fluidic channel design, the amount of solution used in the opto–FET system was significantly less compared with that used in the UV–Vis system. Our fluidic system can typically handle high fluid volumes of approximately 14.1 μL; yet it can provide improved sensitivity and accuracy within a few minutes. Therefore, with its advantages, the opto-FET system can be incorporated into existing bio–NWFET sensors, making it a dual-function detection platform for biomolecules.

## 4. Discussion

An optical biosensing system based on a bio-NWFET has been presented. The entire system was assembled in a compact prototype that demonstrated good photoresponse over a broad wavelength range. The molecular absorption of the quercetin–Cu^2+^ complex was adopted as the basis for biomedical application in our system. Detection of the molecular absorption of metal complexes was performed for Cu^2+^ concentrations ranging from 0.1 μM to 100 μM, and the system exhibited a linearity and sensitivity detection of 0.1 μM Cu^2+^. The measured absorbance was compared with that obtained from a commercial UV–Vis spectrometer. The opto–FET system offers significant potential for the biotechnology and healthcare industries as an optical biosensing device with high detection capability, high reproducibility, and low sample volume requirements.

## Data Availability

Not applicable.

## Supplementary Materials

The following supporting information can be downloaded at: https://www.mdpi.com/article/10.3390/s22197219/s1, Figure S1: SEM image of the top view of the NW-FET channel area with a scale bar of 4 μm.; Figure S2: The visual change in molecular absorption of quercetin and Cu^2+^. The free quercetin (left) followed by mixing of quercetin and various Cu^2+^concentrations of 1, 10, 20, 40, 60, 80, and 100 μM.
