# Supplementary material for: Opto Field-Effect Transistors for Detecting Quercetin–Cu2+ Complex
Source: Sensors (Basel). 2022 Sep 23;22(19):7219. doi: 10.3390/s22197219 (PMC9573373; doi:10.3390/s22197219)
Supplement: Supplementary file 1 [file sensors-22-07219-s001.zip › sensors-1903860-supplementary.pdf]

Supplementary Materials

Pradhana Jati Budhi Laksana[1,2,3], Li-Chu Tsai[4,*], Chang-Cheng Lin[4], Kuei-Shu Chang-Liao[1], Mathew K. Moodley[5], and Chii-Dong Chen[3,*]

Device Fabrication

NW-FET chips were fabricated using top-down technology on six-inch silicon-on-insulator (SOI) wafers with 375 nm-thick oxides and 100 nm-thick top silicon. The top silicon layer of each SOI wafer was n-type doped with phosphorus at a concentration of $1x10^{11}$ cm^{-2} and acceleration energy of 20 keV to achieve a concentration between 10^{16} cm^{-3} and 10^{18} cm^{-3}. Dopant activation was performed using rapid thermal annealing (RTA) at 1000 °C for 20 s. This layer was etched using conventional optical lithography and inductively-coupled plasma reactive-ion etching (ICP-RIE) to define the sensing region, including the source/drain and nanowire. The sensing area was isolated from the outside environment by a 15 nm-thick silicon oxide grown using a wet oxidation furnace at 900 °C. The sensing area was protected by a 150 nm-thick protective layer. The source and drain electrodes were subjected to additional doping of arsenic to a concentration of $2x10^{14}$ cm^{-2}, followed by a 1000 °C 20 s activation process to reduce contact resistance. An oxide with a thickness of 600 nm was used for the interlayer dielectric (ILD) to prevent crosstalk between adjacent metal lines. A metal stack of 800 nm aluminum on 150 nm titanium was defined using the lift-off process for source/drain contacts. The source/drain line was then passivated by silicon oxide and nitride to avoid a direct connection to the sample solution. The length, width, and thickness of the silicon sensing wire between the source and drain electrodes were 2 μm, 200 nm, and 100 nm, respectively.

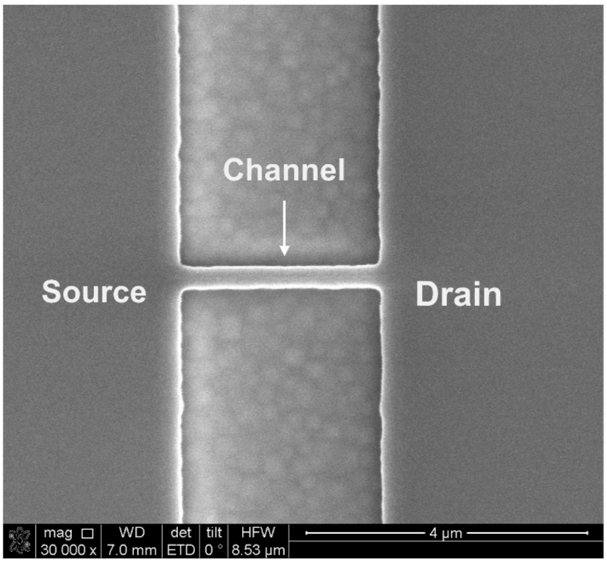

Figure S1. SEM image of the top view of the NW-FET channel area with a scale bar of 4 μm.

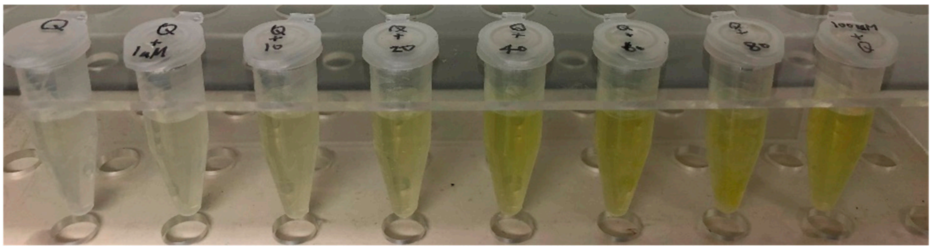

Figure S2. The visual change of molecular absorption of quercetin and Cu^{2+}. The free quercetin (left) followed by mixing of quercetin and various Cu^{2+}concentrations of 1, 10, 20, 40, 60, 80, and 100 μM.
